# Tracing SARS-Coronavirus Variant with Large Genomic Deletion

**DOI:** 10.3201/eid1101.040544

**Published:** 2005-01

**Authors:** Rossa W.K. Chiu, Stephen S.C. Chim, Yu-kwan Tong, Kitty S.C. Fung, Paul K.S. Chan, Guo-ping Zhao, Y.M. Dennis Lo

**Affiliations:** * The Chinese University of Hong Kong, Shatin, Hong Kong SAR; † The Chinese National Human Genome Center at Shanghai, Shanghai, China

**Keywords:** letter, SARS, coronavirus, mutant, epidemiology

**To the Editor:** Severe acute respiratory syndrome (SARS) has been a global public health issue (*1*). We completed a study on the evolutionary path of the SARS-associated coronavirus (SARS-CoV) during the 2002–2003 epidemic (*2*). Most human SARS-CoV strains, as exemplified by the Tor2 sequence (GenBank accession no. AY274119) (*3*), are characterized by the deletion of a 29-nucleotide (nt) segment upstream of the nucleocapsid (N) gene domain when compared with the viral strains isolated from the earliest human SARS patients (*2*) or from nonhuman mammalian hosts (*4*). Towards the end of the epidemic, a variant of the SARS-CoV with a deletion of 386 nt flanking the 29-nt site was first demonstrated by complete genomic sequencing in 2 patients in Hong Kong (GenBank accession nos. AY394999, AY395000, AY395001, AY395002) (*2*). The 386-nt deleted segment corresponds to the genomic region spanning residues 27719 to 28104 of the Tor2 sequence (*3*). The deletion results in the disruption of a putative open reading frames, orf 9, while eliminating orfs 10 and 11. This deletion variant was first isolated from 2 SARS patients with disease onset in mid-May 2003. Patient A was a 41-year-old female phlebotomist working in North District Hospital, New Territories East Cluster, Hong Kong. Patient B was a 98-year-old female admitted to ward X of North District Hospital (*2*).

With this finding late in the epidemic, we studied the prevalence of this SARS-CoV variant to determine its origin. Twenty-one SARS patients with disease onset dates from mid-April were identified. All cases had been confirmed by positive reverse transcription–polymerase chain reaction (RT-PCR) detection of SARS-CoV RNA in clinical specimens or seroconversion. These patients had been admitted with SARS to 4 different hospitals in Hong Kong, including North District Hospital and hospitals A and B, which were located in the same geographic cluster as North District Hospital, as well as hospital C, which was geographically distant from the other 3 hospitals. Clinical specimens were retrieved, and RT-PCR was performed to specifically amplify a genomic segment of SARS-CoV encompassing the deletion site. Specimens with shortened PCR fragments were sequenced to determine the location and precise extent of the deletion.

RT-PCR products were not observed in 2 specimens. Gel electrophoresis of the RT-PCR products for each of the remaining 19 specimens showed a single genomic fragment; 13 of these fragments were shortened. Direct sequencing of the short amplicons showed a deletion of 386 nt identical to that isolated from patients A and B. The patients’ histories were reviewed. Patients A, B, and the 13 patients appeared to be epidemiologically related. The epidemiologic relationships and clinical details of the 15 cases are illustrated in the Figure. Most of the cases were part of a documented outbreak at North District Hospital traceable to an 85-year-old woman, L, in whom SARS was not initially suspected but was subsequently confirmed by seroconversion. Patient L had been admitted to ward Y of North District Hospital; subsequently SARS developed in 7 fellow inpatients (patients 3, 4, 5, 6, 8, D, and E) and 2 healthcare workers (patients 7 and A) (Figure). Patient A had been working in both wards X and Y, and patient D was transferred from ward Y to X before symptom onset. Soon afterwards, SARS developed in 2 other inpatients (patients B and 11) in ward X (Figure). Patient E was transferred from ward Y to Z, where symptoms later developed in another inpatient (patient 2) (Figure).

**Figure:**
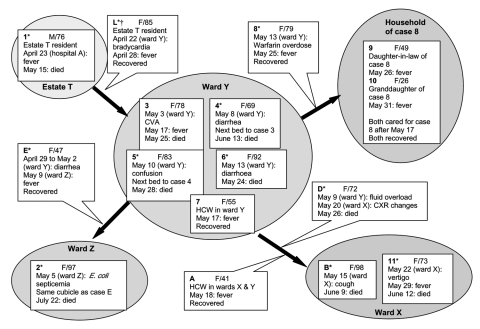
Schematic illustration of the epidemiologic relationships between patients with the severe acute respiratory syndrome-associated coronavirus (SARS-CoV) variant with the 386-nt deletion. Patients are grouped according to the most probable site where SARS infection was acquired. Blocked arrows indicate the potential epidemiologic relationships between subgroups of patients. Patients who are suspected of being an epidemiologic link between particular subgroups are indicated by their association with the respective blocked arrows. For each patient, “F” denotes female, and “M” denotes male, and age is specified. The date of admission, followed by admission site in parentheses, and the initial complaint are indicated next. Additional noteworthy clinical information and the subsequent outcome of each case are indicated. *Patients with a history of chronic illness. †The viral genotype was not characterized in patient L due to the lack of clinical specimens positive for SARS-CoV by reverse transcription–polymerase chain reaction. CVA, cerebrovascular accident; HCW, healthcare worker; E. coli, *Escherichia coli*.

Patients 1, 9, and 10 had not been admitted to North District Hospital but were admitted directly to hospital A. Patients 9 and 10 were household contacts of patient 8 (Figure). Patient 1 had no documented contact with other SARS patients, but coincidentally, patients 1 and L resided in the same estate, T, where a cluster of SARS cases had been documented by the local government (*5*). The deletion variant was absent in 6 of the studied cases. These case-patients had no identifiable relationship with the cohort of patients illustrated in the Figure and did not reside in the same geographic region as patients L and 1. Three of the patients were admitted to and treated in hospital C. None of the 6 patients had been admitted to North District Hospital.

Therefore, we have isolated a SARS-CoV variant with the largest genomic deletion reported to date in a total of 15 SARS patients, 14 females and 1 male, with ages ranging from 26 to 98 years (median 73 years) (Figure). Nine (60%) of the 15 patients, 8 of whom were known chronic disease patients, died (Figure). This mortality rate is consistent with previous observations where the death rate in patients >65 years generally exceeded 50% (*1*). Despite the disruption of several putative orfs, as evident from this study, this SARS-CoV variant remained effective in propagating among persons, particularly in the healthcare setting. The predicted orfs 9, 10, and 11 of the SARS-CoV thus may not be functionally important, although further studies are required. We were able to document 3 generations of transmission and traced the first appearance of this deletion variant to mid-April 2003, possibly at Estate T. Investigation on the origin of this enigmatic variant should be continued by studying its prevalence among the earlier human SARS-CoV isolates and potential mammalian hosts.
